# Bed Bug Infestations and Control Practices in China: Implications for Fighting the Global Bed Bug Resurgence

**DOI:** 10.3390/insects2020083

**Published:** 2011-04-11

**Authors:** Changlu Wang, Xiujun Wen

**Affiliations:** 1 Department of Entomology, Rutgers University, New Brunswick, NJ 08901, USA; E-Mail: cwang@aesop.rutgers.edu; 2 Forest College, South China Agricultural University, 483 Wushan Road, Tianhe, Guangzhou, 510642, China

**Keywords:** *Cimex lectularius*, *Cimex hemipterus*, infestation, control, survey

## Abstract

The bed bug resurgence in North America, Europe, and Australia has elicited interest in investigating the causes of the widespread and increasing infestations and in developing more effective control strategies. In order to extend global perspectives on bed bug management, we reviewed bed bug literature in China by searching five Chinese language electronic databases. We conducted telephone interviews of staff from 77 Health and Epidemic Prevention Stations in six Chinese cities in November 2010. We also conducted telephone interviews of 68 pest control firms in two cities during March 2011. Two species of bed bugs (*Cimex lectularius* L. and *Cimex hemipterus* (F.)) are known to occur in China. These were common urban pests before the early1980s. Nationwide “Four-Pest Elimination” campaigns (bed bugs being one of the targeted pests) were implemented in China from 1960 to the early 1980s. These campaigns succeeded in the elimination of bed bug infestations in most communities. Commonly used bed bug control methods included applications of hot water, sealing of bed bug harborages, physical removal, and applications of residual insecticides (mainly organophosphate sprays or dusts). Although international and domestic travel has increased rapidly in China over the past decade (2000–2010), there have only been sporadic new infestations reported in recent years. During 1999–2009, all documented bed bug infestations were found in group living facilities (military dormitories, worker dormitories, and prisons), hotels, or trains. One city (Shenzhen city near Hong Kong) experienced significantly higher number of bed bug infestations. This city is characterized by a high concentration of migratory factory workers. Current bed bug control practices include educating residents, washing, reducing clutter, putting items under the hot sun in summer, and applying insecticides (pyrethroids or organophosphates). There have not been any studies or reports on bed bug insecticide resistance. Difficulties of control were noted in our surveys of dormitories in which crowded living, seasonal worker migration, and financial constraints contributed to control failures. This study supports the following conclusions: (1) the bed bug infestation in China dramatically decreased following the campaigns from 1960 to the early 1980s; (2) In our survey of Health and Epidemics Prevention Stations, no bed bug cases were reported in Beijing and Shanghai for the past 12 months, but complaints were reported in Guangzhou, Lanzhou, Urumqi, and Shenzhen; (3) Current bed bug infestations primarily are reported in crowded living environments or transient environments such as worker dormitories and military dormitories. These findings suggest that community-wide bed bug monitoring and control campaigns are necessary for effective control of bed bug infestations as a societal response.

## 1. Introduction

The bed bug (*Cimex lectularius* L.) (also called “common bed bug”) and the tropical bed bug (*Cimex hemipterus* (F.)) are both pests that were very common in the recent past. They became rare with the advent of modern pesticides but have reemerged as common pests in many industrialized countries [1–5]. The dramatic increase in the number of *C. lectularius* infestations has raised significant public attention through media exposure in the U.S., Canada, Australia, and some European countries with increasing alarm. In addition to this media attention, government agencies have taken notice under public pressure and formed coalitions, working parties and task force advisory groups to formulate guidelines and best practices to stop the spread of bed bugs and effectively manage infestations. International travel, changes in pest control practices, stigma attached to the reporting of bed bugs, and resistance to insecticides have been highlighted as contributing factors to the current global bed bug resurgence [1] but it is not clear why only some countries are experiencing bed bug resurgence. Our understanding of why the resurgence has not occurred in some countries in relation to control practices and specifics of infestations may shed light on the global bed bug resurgence and elucidate control difficulties.

**Figure 1:**
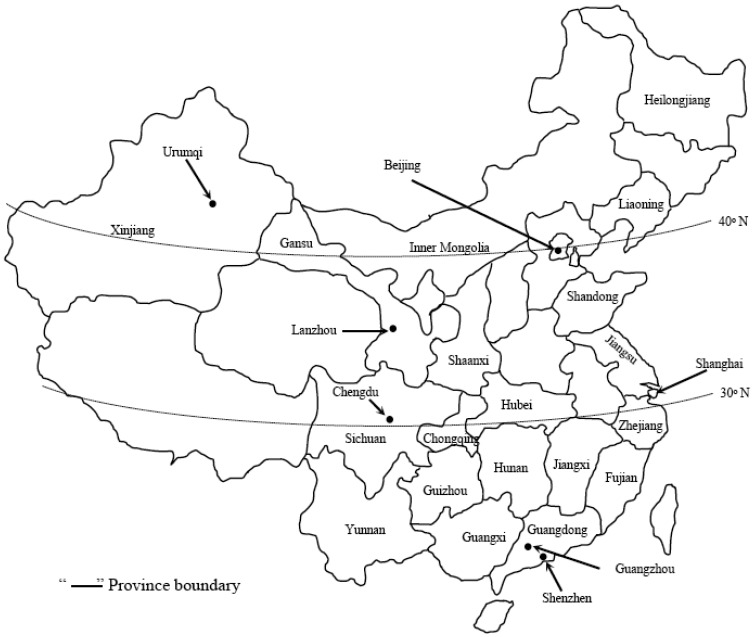
Geographic locations of the surveyed cities.

*C. lectularius* and *C. hemipterus* both occur in China [6]. *C. lectularius* was found from Guangdong, Yunnan, and Fujian provinces in the south to Heilongjiang province in the north (Figure 1). It is most common in areas between 30–40° N in latitude. Its western distribution limit was Chengdu, Sichuan province, based on collections identified by Deng and Feng (1953) [6]. *C. hemipterus* was the dominant species in Guangdong province and Guangxi Zhuang Autonomous Region based on examined specimens [6]. It was also found in Sichuan, Guizhou, Hunan, and Fujian provinces.

Bed bugs were common pests in China before the early 1980s. Ma [7] documented that 9.6% of the residences were infested in Shanghai city in 1979 based on a combination of interviews and visual inspections. Infestations became rare as a result of the national “Four-Pest Elimination” campaign during the period 1960–1980s. The purpose of this paper is to review the history of bed bug infestation in China, to evaluate the present infestation status, control methods and practices currently in use.

## 2. Materials and Methods

### 2.1. Literature Search

The following Chinese online electronic databases were searched in October 2010 using the Chinese key word “bed bug”: (a) China Academic Journal Network Publishing Database, (b) China Doctoral Dissertations Full-text Database, (c) China Master's Theses Full-text Database, (d) China Proceedings of Conference Full-text Database, (e) China Core Newspapers Full-text Database, and (f) China Patent Database. The search yielded a total of 542 citations. All were written in Chinese, with some articles containing English abstracts. Most of the articles were not focused on bed bugs. A total of 113 articles with descriptions about bed bug survey, infestation, control, or disease transmission were reviewed.

### 2.2. Telephone Surveys

In November 2010, we conducted telephone interviews with staff from 77 Health and Epidemic Prevention Stations in 6 cities. All Epidemic Prevention Stations in these cities were surveyed. Geographic locations of the cities are shown in Figure 1. Shanghai and Beijing were selected because they are the largest cities in China, and accommodate most international tourists in China. The other four cities in the survey have reported bed bug infestations in recent years. The interview question was: Has your agency encountered any bed bug infestations or received bed bug complaints within the past 12 months?

In March 2011, we interviewed staff from 34 pest control companies in Shenzhen and 34 pest control companies in Guangzhou. These companies were selected from the members of the pest control associations in these two cities. Only those companies answered our phone calls were included. The staff were asked the following questions (1) how many employees does your company have? (2) did your company encounter bed bug infestations in the past 12 months? (3) what methods did you use to control bed bugs? and (4) what types of environments were infested?

Although efforts were made to reach the most appropriate personnel during interviews, the interviewed personnel may not be most knowledgeable people in their organizations. In addition, none of the stations had a system of recording bed bug complaints due to the rare occurrence of bed bug infestations. Therefore, it is likely that the reported number of stations receiving complaints was lower than the actual numbers.

## 3. Results

### 3.1. History of Bed Bug Infestations in China

Historical bed bug infestation data was not available for the period prior to the establishment of the People's Republic of China in 1949 due to war, political instability, and a severely depressed economy. Bed bugs were common pests in the 1950s and recognized as an important public health pest [8]. In 1960, during the national patriotic public health campaigns, bed bugs were designated by the Chinese government as one of “Four Pests” (rodents, bed bugs, flies, and mosquitoes) that would be targeted for a nation-wide elimination effort until bed bug infestations became rare [9].

In 1978, a “National Four-Pest Elimination Research—Cockroach and Bed Bug Working Team” was formed to conduct research on cockroach and bed bug infestations. This included monitoring, control, disease transmission, biology, *etc.* Table 1 lists the reported bed bug infestation statistics in China. High infestations rates were reported in residences in the late 1970s and early 1980s. Bed bugs were eliminated or reduced to very low numbers by the end of 1980s [10]. In October 1988, the National Patriotic Public Health Campaign Committee issued bed bug control standards which required the percentage of residences or rooms with bed bugs or fresh bed bug feces must be <2% in cities and towns [11]. However, there were no authoritative reports on levels of compliance by the local governments.

The last organized bed bug survey among residences was conducted in 1996 [24]. Table 1 shows that recent bed bug cases were found in crowded living environments (dormitories, prisons, and military bases), hotels, or transportation tool (train). High infestation rates in military dormitories were continuously reported from 1978 to 2007. It is presumed that the crowded environments and frequent occupant turnover were the factors that facilitated the infestations.

### 3.2. Bed Bug Control Methods

The earliest record in China of controlling bed bugs (referred to as wall lice) was found in “Compendium of Materia Medica” by Li Shizhen published in 1758. The Chinese herbs poria (*Wolfiporia extensa* (Peck) Ginns), *Acorus* spp., and *Herba solani* Nigri, were placed around or under beds to repel bed bugs. Other recommendations for repelling bed bugs included burning *Stemona sessilifolia* (Miq.) Miq., *Resina liquidambaris* Hance, buckwheat straw, phellodendron bark, polygonum, horse hoof, mule hoof, crab shell, and arsenate trisulfide. During the period from 1950 to the 1960s, lindane was the most commonly recommended pesticide to control bed bugs [37–39]. During the period from the 1970s until early 1980s, the government sponsored bed bug control campaigns employed liquid formulations of the organophosphate fenthion (0.5–1%) in combination with non-chemical methods for community-wide bed bug elimination [7,13,16,18,40–42]. The insecticide was brushed into bed bug harborages and sprayed onto beds, walls, and other furniture if sprayers were available. These treatment strategies were extremely successful. Much less commonly used insecticides included DDVP spray, phoxim, DDT, and pyrethrins [38,43].

In addition, non-chemical control methods were often used in conjunction with insecticide treatment. These included taking the beds out and dropping them on the ground from a height to shake out bed bugs, soaking items in cold water for 50 hours, pouring hot water into crevices and holes of mattresses, applying hot steam, putting the bed and other infested items under the hot sun in summer, picking bed bugs using needles, and sealing the crevices and holes in walls and floors [38,44–46].

The bed bug control during 1960s–1980s was often organized and supported by the local government agencies (such as Health and Epidemic Prevention Stations) and various levels of neighborhood organizations. Residents were instructed on how to use non-chemical and chemical methods (mostly fenthion) to eliminate bed bugs. Insecticides were supplied to residents or designated personnel. The control results were evaluated by the government. Follow-up treatments were implemented until elimination of the infestation was achieved [12]. The campaign was usually carried out at the same time period in each neighborhood. Most infestations were eliminated after 2–3 treatments.

The pesticides used since the late 1980s were much more diverse. Chemicals that were commonly used included the organophosphates fenthion [36,47] and DDVP [25–27,48] as well a number of pyrethroid sprays including deltamethrin, cypermethrin, permethrin and cyphenothrin [22,23,28,29,31–33,46,47,49,50]. A liquid mixture (0.75% propoxur, 0.75% deltamethrin, 0.3% cypermethrin) was also available for bed bug control [28]. The insecticides were applied using sprayers, brushes, or fogging machines. Most treatments involved both crack and crevice spray and surface applications. There was little research on non-chemical control techniques, partly due to the excellent control results from chemical methods.

The telephone survey of pest control companies revealed 26 out of 68 companies treated bed bug infestations during the past 12 months. Among those companies encountering bed bugs, five used pyrethroids only, three used both pyrethroids and organophosphates, two used organophosphates only. The rest of the companies did not specify the class of chemicals they used. In addition to chemical treatments, the companies asked clients to place infested clothing and other items under the hot sun and to wash infested items.

### 3.3. Current Bed Bug Infestations

Since 2005, only sporadic bed bug infestations have been reported. The infested environments included hotels [34,51,52], military dormitories [33,34], worker dormitories [31,32] and trains [35,36]. A hotel in Urumqi city serving international guests was infested with bed bugs in 2005 [51]. The infestation was thought to have originated from an introduction by international tourists. The infestation was eliminated within two weeks after application of a combination of cypermethrin and propoxur spray. Dozens to more than one hundred workers living in three construction sites in Beijing were bitten with bed bugs in 2006 and 2007, respectively [31,32]. Xu [53] reported that the organizing committee of the 2008 Olympic Games in Beijing received bed bug complaints from hotel guests. The author also reported a bed bug infestation complaint from factory workers in a company located at Qingdao city, Shandong province. Trains servicing the route from Lanzhou to Chengdu and from Beijing to Chongqing were also reported to be infested.

The telephone interviews of Health and Epidemics Prevention Stations revealed that Shenzhen city had much higher bed bug infestation frequency than other cities (Table 2). All of the Health and Epidemic Prevention Stations of this city reported receiving bed bug complaints. In contrast, only one out of 13 stations in the much larger Guangzhou city (approximately 150 km from Shenzhen) reported bed bug incidences. In Shenzhen city, bed bug infestations were mostly found in migratory worker dormitories. There were some cases among households but few cases in schools, college dormitories, or hotels.

Surveys of 68 pest control companies revealed the median (minimum, maximum) number of employees per company in Shenzhen and Guangzhou were 11 (5, 12) and 15 (5, 110), respectively. The percentages of companies that encountered bed bug infestations were 58.8 and 17.6%, respectively. The infested environments (number of reported companies) were: worker dormitories (12), residences (2), rented rooms (2), and hotel (1). As bed bug specimens were not available from the bed bug incidences, it is not clear whether one or two bed bug species were involved in these reported infestations.

## 4. Discussion

Our survey indicates that bed bugs are currently not a significant concern in China as a result of the national “Four-Pest Elimination” campaigns using a combination of insecticides (organophosphates and/or pyrethroids) and non-chemical control methods (cleaning, pouring hot water, and placing infested items under the hot sun, *etc.*). Although the non-chemical methods being practiced were not likely 100% effective [54], the addition of these methods was considered helpful in the control programs. Community-wide action and government regulation played a significant role in suppressing bed bug infestations to very low levels. The availability of more toxic organophosphate and carbamate insecticides with different modes of action than pyrethroids may also have contributed to the control success.

There were no reports on bed bug insecticide resistance in China. This is in contrast with the report of widespread resistance to DDT during the 1950s [55] and a recent report of prevalence of pyrethroid insecticide resistant bed bug populations in the U.S. [56]. First, the lack of reported resistance may be due to the fact that bed bugs were usually totally eliminated in a short period of time as a consequence of the community-wide control campaigns. Second, the use of both registered organophosphate and pyrethroid insecticides may have prevented bed bugs from developing high levels of resistance. Third, organochlorine insecticides were much less used compared with organophosphates. Fourth, low level resistance might be present when chronic infestations occur (such as in worker dormitories), but was not recognized. Unsatisfactory control was reported from several factories in Shenzhen where worker dormitories had bed bugs for several years (Liu, personal communication).

During the bed bug control campaigns, pesticides were often applied by residents or people with very limited training. The quantity of pesticide used and the areas treated (*i.e.*, surface treatments on furniture or wall) might have posed significant risks to the inhabitants. There were no studies on the levels of pesticide residues resulting from bed bug control and their potential risks to residents. Although there were no complaints or reports on insecticide residues, it is prudent to limit the amount of insecticide use and limit the insecticide use to properly trained personnel. Considering the close contact between residents and the areas of insecticide treatment, research must be done to investigate the potential environmental and health risks of insecticide applications in bed bug management programs. Such research will provide basis for selecting proper insecticides and application techniques for controlling bed bugs.

Infestations in China are mostly found in factory worker or military dormitories at the present time. Common challenges among these communities are: crowded living, frequent turnover, and lack of pest control budget or attention to the issue [57]. Similar challenges exist in multi-unit dwellings occupied by low-income people in the U.S. [58]. The commonality of the challenges exhibited in the two countries suggests that: (1) control failure is related to lack of attention or cooperation; (2) it is necessary to implement periodic community-wide bed bug control campaigns until infestations are eliminated; and (3) government involvement in bed bug control is needed to prevent bed bugs from resurging. The success of bed bug control campaigns conducted in China during the period from the 1960s to early 1980s demonstrates the effectiveness of government organized community-wide bed bug control campaigns.

This study revealed that Shenzhen city is experiencing higher number of bed bug infestations than other cities. This city is densely populated (4,239 person/km^2^ in 2005) and probably has the highest concentration of migrant workers from villages or small towns in China. The crowded living environments, frequent turnovers of occupants, and lack of attention from dormitory management offices enables bed bugs to persist and proliferate. Once established, bed bugs can spread rapidly within a building [58,59]. They can also spread within and between communities if not effectively controlled. For example, bed bug complaints registered by The New York City Department of Housing Preservation and Development rose from only 537 in 2004 to 12,786 in 2010 [60]. Factory workers usually visit their home villages and towns at least once each year. Infestations in worker dormitories therefore are potential reservoirs for spread resulting in new infestations in other cities or villages. Lacking more effective control, it is highly possible that bed bugs may spread into other communities and cause resurgences as we have seen in New York City and other countries. It is therefore urgent for the local government to monitor the distribution and level of bed bug infestations, and to take proactive approach in preventing and eliminating bed bugs in vulnerable communities.

## 5. Conclusions

This study supports the following conclusions: (1) the bed bug infestation in China dramatically decreased following the campaigns from 1960 to the early 1980s; (2) in our survey of Health and Epidemics Prevention Stations, no bed bug cases were reported in Beijing and Shanghai for the past 12 months, but complaints were reported in Guangzhou, Lanzhou, Urumqi, and Shenzhen; and (3) current bed bug infestations primarily are reported in crowded living environments or transient environments such as worker dormitories and military dormitories.

## Figures and Tables

**Table 1:** Summary of reported bed bug infestation statistics in China.

**Location**	**Structure Type**	**Survey Year**	**Number of Units Surveyed**	**Number or % with Bed Bugs**	**Reference**
Nanshi district, Shanghai	Residences	1979–1985	17,3198 to 21,6028 per year	From 9.6124% in 1979 to 0.0093% in 1985	[7]
Huangpu district, Shanghai	Residences	1982	145,695	303	[12]
Nanjing, Jiangsu province	Residences	1981–1982	283,018	4.78%	[13]
Dandong city, Liaoning province	Residences	1980–1981	1,843	49	[14]
Urumqi, Xinjiang Uyghur Autonomous Region	Residences	1981	1,060	213	[15]
Fushun, Liaoning province	Residences	1981	64,213	2,433	[16]
Yunnan province	Military dormitories	1982	104,146 beds	5,170 beds	[17]
Lengshuitan, Hunan province	Residences	1982	9,459	1.20%	[18]
Lengshuitan, Hunan province	Residences	1984	10,051	0.12%	[18]
Lengshuitan, Hunan province	Residences	1985	11,512	0.07%	[18]
Fengcheng, Liaoning province	Residences	1988–1989	23,865	0	[19]
Fengcheng, Liaoning province	Hotels, public rooms	1988–1989	2,741 rooms	0	[19]
Fujian province	Military dormitories	1990	450 beds	415 beds	[20]
Hubei province	Hotels	1992	78	5	[21]
Hubei province	Hospitals	1992	47	13	[21]
Hubei province	Schools	1992	3	1	[21]
Hubei province	Residences	1992	6,614	413	[21]
Huhehaote, Inner Mongolia	School	1990?	69 rooms	36.2%	[22]
Zhejiang province	Residences	1992	51,870	8	[23]
Jiangshan, Zhejiang province	Residences	1996	5,228	0	[24]
Nanjing, Jiangsu province	A prison	1999	32 beds	32 beds	[25]
Jiangxi province	Worker dormitories	2000	24 rooms	24 rooms	[26]
Shaanxi province	College dormitories	2001–2002	720 beds	16 beds	[27]
Nanjing, Jiangsu province	A prison	2002–2003	92 beds	77 beds	[28]
Jinan, Shandong province	Military dormitories	2002	124 beds	63 beds	[29]
Shaanxi province	Military dormitories	2002	1,183 beds	108 beds	[30]
Beijing	Worker dormitories	2006	36 beds	34 beds	[31]
Beijing	Worker dormitories	2007	Unknown	>100 workers	[32]
Jinan, Shandong province	Military dormitories	2007	156 beds	50 beds	[33]
Jinan, Shandong province	A hotel	2006	Unknown	8 rooms	[34]
Jinan, Shandong province	A military dormitory	2006	Unknown	85% of the rooms	[34]
Beijing – Chongqing	Trains	2008	17 train cars	3 train cars	[35]
Hubei province	Trains	2009	116 train cars	3 train cars	[36]

**Table 2:** Bed bug complaints received by Health and Epidemics Prevention Stations in six Chinese cities.

**City**	**Number of Stations Surveyed**	**Number of Stations Received Bed Bug Complaints**
Beijing	20	0
Guangzhou	13	1
Lanzhou	8	2
Shanghai	20	0
Shenzhen	8	8
Urumqi	8	3
